# Antioxidant, Lipid Lowering, and Membrane Stabilization Effect of Sesamol against Doxorubicin-Induced Cardiomyopathy in Experimental Rats

**DOI:** 10.1155/2013/934239

**Published:** 2013-10-21

**Authors:** Anusha Chennuru, Mohamed T. S. Saleem

**Affiliations:** Department of Pharmacology, Annamacharya College of Pharmacy, Rajampet, Andhrapradesh 516126, India

## Abstract

The present study was designed to evaluate the cardioprotective effect of sesamol against doxorubicin-induced cardiomyopathy in rats. In this study, the cardioprotective effect of sesamol against doxorubicin induced cardiomyopathy in experimental rats was evaluated at the dosage of 50 mg/kg bw. Doxorubicin was administered to rats at a total cumulative dose of 15 mg/kg through intraperitoneal route for 2 weeks in six-divided dose on 8th, 10th, 14th, 16th, 18th, and 21st day. After the last dose administration, the endogenous antioxidants and lipid peroxidation were estimated in heart tissue homogenate. Cardiac biomarkers such as troponin T, LDH, CK, and AST and lipid profiles such as cholesterol, triglycerides, HDL, LDL, and VLDL were estimated in serum. Sesamol has cardioprotective activity through normalization of doxorubicin-induced-altered biochemical parameters. Biochemical study was further supported by histopathological study, which shows that sesamol offered myocardial protection from necrotic damage. From these findings, it has been concluded that the sesamol has significant cardioprotection against doxorubicin induced cardiomyopathy via amelioration of oxidative stress, lipid lowering, and membrane stabilization effect.

## 1. Introduction

Doxorubicin (DOX) is one of the most efficient anticancer antibiotics. The clinical use of DOX is limited due to the extensive adverse effects. Chronic administration of DOX to cancer patients causes dose-dependent cardiotoxicity which leads to heart failure and cardiomyopathy [1, 2]. It is reported that 41% of cancer patients who received DOX are affected with various cardiac problem. DOX treatment increases the morbidity and mortality of cancer patients due to the heart failure [3]. 

The heart is distinctively susceptibility to oxidative damage. DOX-induced cardiomyopathy is strongly linked to an increase in cardiac oxidative stress, as indicated by the depletion of endogenous antioxidant enzymes, and accumulation of free radicals in the myocardium which increases the chance of DOX-induced cardiomyopathy [4]. Several therapeutic interventions implemented to protect the heart from DOX-induced cardiomyopathy. However, higher mortality through abnormal cardiac activity limited the ability of protective role of these therapies [2]. So the search of novel molecule to ameliorate the DOX-induced cardiotoxicity is exceedingly urgent. Administration of antioxidant drugs to protect the heart from free radical damage getting more attention in cardiovascular disease research [1].

Sesamol is a potent phenolic antioxidant which is a component of sesame oil. It is white crystalline powder, sparingly soluble in water, and miscible with most of oils. Antioxidant property of sesamol has been shown earlier to exhibits radioprotective [5], antimutagenic [6], gastroprotective [7], neuroprotective [8], and antiplatelet activity [9]. It is reported that administration of sesamol protect the myocardium from isoproterenol-induced myocardial injury via antioxidative mechanism [10]. In the light of the above literature, the present study was undertaken to evaluate the effect of sesamol on DOX-induced cardiomyopathy.

## 2. Materials and Methods

### 2.1. Chemicals

Sesamol and doxorubicin was purchased from Sigma-Aldrich, India. All chemicals were of analytical grade purchased from Sigma-Aldrich, India. 

### 2.2. Animals

Healthy albino Wistar rats of either sex weighing between 180–200 g of 3 months of age were used. Animals were housed individually in polypropylene cages, maintained under standard conditions (12:12 L:D cycle; 25 ± 3°C; and 35–60% humidity), and fed with standard rat pellet diet (SaiDurga Feeds and Foods, Bangalore) and water *ad libitum*. The study protocol was approved by the Institutional Animal Ethics Committee (File number ANCP/IAEC/01-2013).

### 2.3. Experimental Protocol

After 1 week of acclimatization, the animals were randomly divided into four groups with six animals in each group. Group 1 (G1) served as normal control and received normal saline (10 mL/kg body weight p.o.). Group 2 (G2) received sesamol 50 mg/kg body weight i.p. (dose selected based on previous report by Vennila and Pugalendi [10]) for 7 days and then alternatively with vehicle for the next 2 weeks. Group 3 (G3) was treated with DOX (total cumulative dose of 15 mg/kg i.p. for 2 weeks in six divided dosage on 8th, 10th, 14th, 16th, 18th, and 21st day). Group 4 (G4) was pretreated with sesamol 50 mg/kg body weight i.p. for 7 days followed by DOX administration as in G3.

All animals from respective groups were observed for heart weight, body weight changes, and mortality. After 24 h of last treatment, rats were anaesthetized with pentobarbitone sodium (60 mg/kg^−1^), and serum was separated from the blood for the estimation of cardiac biomarkers like troponin-T, lactate dehydrogenase (LDH), creatinine kinase (CK), and aspartate transaminase (AST) and lipid parameters like total cholesterol (TCH), triglyceride (TGL), low density lipoprotein (LDL), very low density lipoprotein (VLDL), and high density lipoprotein (HDL) by using enzyme kits (Transasia Bio-Medicals Limited, Solan). Animals from respective groups were sacrificed, and the heart tissue was quickly dissected, washed in ice cold saline, dried on filter paper, and weighed immediately. A portion of each heart was taken from all the groups, and homogenate was prepared in 0.3 M phosphate buffer (pH 7.4) for the estimation of thiobarbituric acid reactive substance (TBARS) [11], reduced glutathione (GSH) [12], superoxide dismutase (SOD) [13], catalase [14], and protein [15]. 

### 2.4. Histopathological Examination

The heart was isolated and washed immediately with ice cold saline then fixed in 10% formalin. After fixation tissues were embedded in paraffin-wax, and thick sections were cut into thin sections and stained with hematoxylin and eosin. These slides were then observed under light microscope for histopathological changes. 

### 2.5. Statistical Analysis

Values are expressed as mean ± SD and analyzed using Graph Pad prism version 5.1 using ANOVA fallowed by Tukey's multiple comparison test. *P* < 0.05 was considered significant.

## 3. Results

There was no mortality observed in any of the treatment group.

### 3.1. Effect of Sesamol on Heart Weight : Body Weight Ratio (1 × 10^−3^)

**Figure 1:**
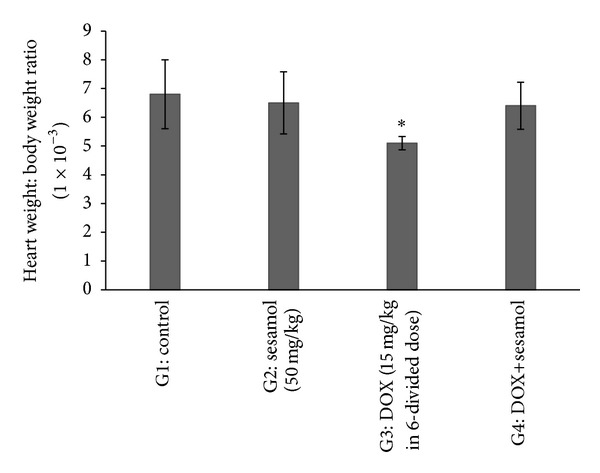
Effect of sesamol on heart weight : body weight ratio. *P < 0.05 versus G1.

There was a significant (*P* < 0.05) decrease in the heart weight : body weight ratio in DOX treated group (G3) compared to control group (G1). There was no significant fall in the heart weight : body weight ratio in sesamol alone (G2) and sesamol+DOX (G4) treated groups compared to G3 (Figure 1).

### 3.2. Effect of Sesamol on Serum Lipid Profile

There was no significant difference in the level of TCH, TGL, LDL, VLDL and HDL in sesamol treated group (G2) when compared to control group (G1). There was significant (*P* < 0.05) increase in the level of TCH, TGL, LDL, and VLDL and significant (*P* < 0.05) decrease in the level of HDL in DOX (G3) treated group when compared to the control group. There was significant (*P* < 0.05) decrease in the level of TCH, TGL, LDL, VLDL and significant (*P* < 0.05) increase in the level of HDL in sesamol+DOX (G4) treated group when compared to the DOX treated group (Table 1).

### 3.3. Effect of Sesamol on Serum Troponin-T, LDH, CK, and AST

There was no significant difference in the level of troponin-T, LDH, CK, and AST between control (G1) and sesamol (G2) alone treated rats. Rats that were treated with DOX (G3) significantly (*P* < 0.0001) increased the level of troponin-T, LDH, CK, and AST when compared with control (G1). Rats that were pretreated with sesamol followed by DOX (G4) (*P* < 0.0001) significantly decreased the level of troponin-T, LDH, CK, and AST when compared with DOX (G3) treated rats (Table 2). 

### 3.4. Effect of Sesamol on TBARS

There was no significant difference in the level of TBARS between control (G1) and sesamol (G2) alone treated rats. Rats that were treated with DOX (G3) significantly (*P* < 0.0001) increased the level of TBARS when compared with control (G1). Rats that were pretreated with sesamol followed by DOX (G4) (*P* < 0.001) significantly decreased the level of TBARS when compared with DOX (G3) treated rats (Table 3).

### 3.5. Effect of Sesamol on GSH, SOD, and Catalase

There was no significant difference in the level of GSH and SOD between control (G1) and sesamol (G2) alone treated rats, and there was significant (*P* < 0.0001) difference in the level of catalase in sesamol (G2) alone treated rats when compared to control (G1) group. Rats that were treated with DOX (G3) significantly (*P* < 0.0001) decreased the level of GSH, SOD, and catalase when compared to control (G1). Rats were pretreated with sesamol followed by DOX (G4) significantly (*P* < 0.0001) decreased the level of GSH, SOD, and catalase when compared to DOX (G3) treated rats (Table 3). 

### 3.6. Histopathology

**Figure 2:**
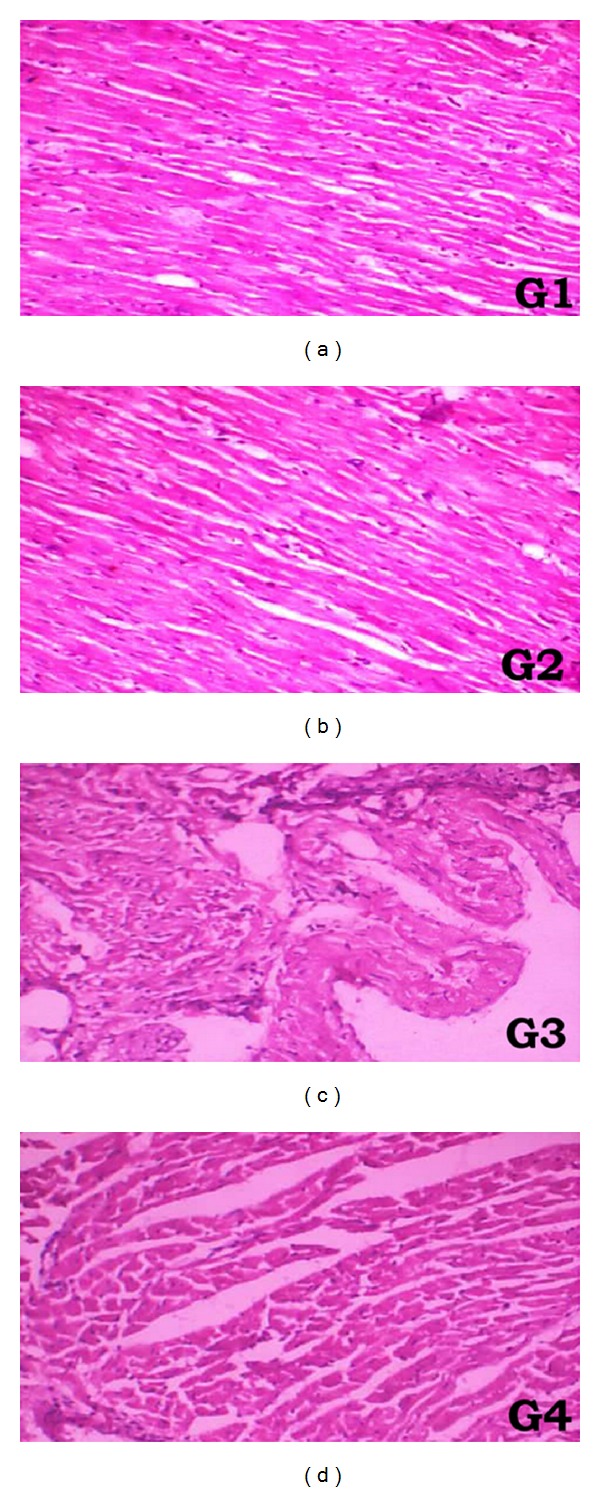
Light microscopical analysis of heart tissue: (G1) heart of rat received saline, (G2) heart of rat received sesamol, (G3) heart of rat received DOX, and (G4) heart of rat received sesamol+DOX.

Light microscopical section of heart tissue (Figure 2) of saline treated rat (G1) and sesamol treated rat (G2) show normal architecture of myocardium. Rats treated with DOX (G3) shows severe myocardial necrosis with subendocardial loss of muscles. Rats treated with sesamol+DOX show well-preserved myocardium when compared to DOX (G3) treated group.

## 4. Discussion

The present study endows with the significant indication of the beneficial effects of sesamol on DOX-induced cardiomyopathy. The outstanding remarkable findings were that the sesamol prevented the DOX-induced-altered lipid parameters, myocardial marker enzymes, and antioxidant parameters. Our findings suggest that sesamol protects the myocardium through numerous aspects against DOX-induced cardiomyopathy.

Generation of reactive oxygen species (ROS) is one of the mechanism for DOX-induced myocardial toxicity. DOX metabolically converted into its semiquinone form by CYP450 and flavin monooxygenase enzymes. This metabolite interacts with mitochondrial oxygen to generate ROS. In another way DOX reacts with iron (Fe^2+^) to make free radical complex, which involves in the generation of hydroxyl radical (OH^∙^) [16, 17]. 

Oxidative stress plays major role in DOX-induced cardiotoxicity by generation of lipid peroxidation. Myocardial tissue susceptible to free radical damage due to less amount of antioxidants like SOD and catalase present in the heart [18, 19]. Administration of DOX at cumulative dose (15 mg/kg) increases the lipid peroxidation and depleted the endogenous antioxidants in the myocardium. Similar biochemical changes have been reported by several other studies [20, 21]. In the present study, generation of lipid peroxidation is conformed by elevated level of TBARS in DOX administered rat. Elevated level of TBARS significantly decreased by sesamol indicated the protective role of sesamol via reduction of oxidative stress. Moreover, sesamol that enhanced the GSH, SOD, and catalase in the myocardium supported the antioxidative effect of sesamol against DOX-induced oxidative damage. In the present study we have observed the putative antioxidant property of sesamol against DOX-induced cardio toxicity. Previous studies have demonstrated that sesamol exhibits antioxidant property in various oxidative conditions that cause tissue injury [10, 22–24]. 

Generation of free radical extensively damage the myocardium result in increased membrane permeability leads to leakage of LDH, CK and AST [25]. In the present study, sesamol significantly decreases the elevated level of serum LDH, CK, and AST. These results were consistent with the previous studies reported by other investigators [25, 26]. Another important findings observed in the present study are that sesamol significantly decreases the elevated level of serum troponin-T. Troponins are myocardial regulatory proteins, which regulate the calcium mediated actin and myosin interaction. Troponin-T is widely used as specific marker to diagnose myocardial infarction. Sesamol pretreatment significantly decreased the elevated level of serum troponin-T near to normal level which conformed the membrane stabilizing effect of sesamol against DOX-induced myocardial damage. 

Lipids plays important role in cardiovascular disease complications. Drugs with lipid lowering agent protect the myocardium from DOX-induced cardiotoxicity [27]. DOX interferes with metabolism and biosynthesis of lipids there by TGL, TCH, LDL, VLDL levels were increased and HDL level were decreased in serum. In the present study, we have observed that the DOX significantly increased the level of TGL, TCH, LDL, and VLDL and decreased the level of HDL. Sesamol pretreatment that significantly reverted these lipid parameters near to normal indicated that sesamol may be lowering the lipids due to the inhibition cholesterol biosynthesis and increase in the uptake of LDL from blood by liver [3]. Similar effect was observed by Vennila and Pugalendi [28] in isoproterenol-induced myocardial infarction. 

The biochemical data was supported by histopathological report, which showed severe myocardial necrosis with subendocardial loss of muscles in DOX administered rat. Sesamol shows that well-preserved myocardium was the indication of cardio protection. 

Nayak et al. [29] reported the cardioprotective effect of sesamol against DOX-induced cardiotoxicity by *in vitro *experimental model. In this study they have reported that sesamol pretreatment increased the antioxidant status by preventing free radical generation. Similar effect has been observed in the present research conformed the significant effect of sesamol in ameliorating the toxic effects of DOX associated with cancer chemotherapy. 

## 5. Conclusion

From these findings it has been concluded that our study, for the first time, demonstrates that sesamol protects the myocardium from DOX-induced cardiomyopathy in rat. The protective role may be due to its antioxidative, lipid lowering, and membrane stabilizing effect.

## Figures and Tables

**Table 1:** Effect of sesamol on serum TCH, TGL, LDL, VLDL, and HDL.

Group	TGL (mg/dL)	TCH (mg/dL)	LDL (mg/dL)	VLDL (mg/dL)	HDL (mg/dL)
G1: control	103.0 ± 5.8	103.0 ± 1.7	51.5 ± 2.2	20.5 ± 0.76	26.3 ± 0.42
G2: sesamol (50 mg/kg)	105.0 ± 2.2	110.8 ± 2.9	56.0 ± 3.2	19.5 ± 0.76	27.5 ± 0.76
G3: DOX (15 mg/kg in 6-divided dose)	169.0 ± 4.4^a^	131.3 ± 3.4^a^	73.5 ± 2.2^a^	28.0 ± 3.2^a^	19.0 ± 3.0^a^
G4: DOX+sesamol	79.00 ± 2.2^b^	110.0 ± 2.0^b^	62.5 ± 0.76^b^	20.5 ± 0.76^b^	27.6 ± 0.42^b^

All values expressed as mean ± SEM. ^a^ *P* < 0.05 versus G1. ^b^ *P* < 0.05 versus G3.

**Table 2:** Effect of sesamol on serum LDH, CK, AST, and troponin-T.

Group	LDH IU/L	CK IU/L	AST IU/L	Troponin-T *µ*g/mL
G1: control	109.2 ± 6.493	114.3 ± 6.766	121.5 ± 2.9	0.50 ± 0.21
G2: sesamol (50 mg/kg)	119.0 ± 2.309	128.3 ± 1.7	138.0 ± 0.89	0.59 ± 0.34
G3: DOX (15 mg/kg in 6-divided dose)	299.0 ± 20.67^a^	249.3 ± 4.2^a^	345.0 ± 17.8^a^	1.7 ± 0.63^a^
G4: DOX+sesamol	107.5 ± 5.696^b^	185.7 ± 0.88^b^	119.0 ± 16.9^b^	0.81 ± 0.14^b^

All values expressed as mean ± SD. ^a^ *P* < 0.0001 versus G1. ^b^ *P* < 0.0001 versus G3.

**Table 3:** Effect of sesamol on TBARS, GSH, SOD, and catalase.

Groups	TBARS (nmol/g wet wt)	GSH (*µ*g/g wet wt)	SOD (IU/mg protein)	CAT (IU/mg protein)
G1: control	6.9 ± 1.7	227.7 ± 6.2	102.2 ± 3.7	33.3 ± 2.2
G2: sesamol (50 mg/kg)	6.3 ± 1.2	237.9 ± 10.3	103.8 ± 6.04	38.8 ± 1.3***
G3: DOX (15 mg/kg in 6-divided dose)	15.8 ± 2.8^a^	130.2 ± 2.9^a^	47.7 ± 8.27^a^	22.09 ± 1.9^a^
G4: DOX+sesamol	11.07 ± 1.8^b^	150.5 ± 5.8^b^	94.02 ± 5.5^b^	29.3 ± 1.9^b^

All values expressed as mean ± SD. ***P < 0.0001 versus G1, ^a^ *P* < 0.0001 versus G1, and ^b^ *P* < 0.001 versus G3.
